# Instrumental activities of daily living trajectories and risk of mild cognitive impairment among Chinese older adults: results of the Chinese longitudinal healthy longevity survey, 2002–2018

**DOI:** 10.3389/fpubh.2023.1165753

**Published:** 2023-05-03

**Authors:** Jialu Yang, Yangchang Zhang, Shisi Shen, Han Yu, Luran Yang, Yao Zhao, Yang Xiong, Jiayi Su, Lianlian Wang, Xun Lei

**Affiliations:** ^1^School of Public Health and Management, Chongqing Medical University, Chongqing, China; ^2^Research Center for Medicine and Social Development, Chongqing Medical University, Chongqing, China; ^3^The Innovation Center for Social Risk Governance in Health, Chongqing Medical University, Chongqing, China; ^4^Research Center for Public Health Security, Chongqing Medical University, Chongqing, China; ^5^The First School of Clinical Medicine, Chongqing Medical University, Chongqing, China; ^6^The Second School of Clinical Medicine, Chongqing Medical University, Chongqing, China; ^7^The Second Affiliated Hospital of Chongqing Medical University, Chongqing, China; ^8^The West China Hospital, Sichuan University, Chengdu, China; ^9^The Jiang Jin Central Hospital of Chongqing, Chongqing, China; ^10^Department of Reproductive Center, The First Affiliated Hospital of Chongqing Medical University, Chongqing, China; ^11^State Key Laboratory of Maternal and Fetal Medicine of Chongqing Municipality, Chongqing Medical University, Chongqing, China

**Keywords:** CLHLS, GBTM, IADL, MCI, Chinese older adults

## Abstract

**Background:**

The association between the instrumental activities of daily living (IADL) score and the risk of initial cognitive function impairment is inconclusive. We aimed to identify distinctive IADL trajectories and examine their relationship with the onset of mild cognitive impairment (MCI) among Chinese older people.

**Methods:**

The study used six-wave longitudinal data from the Chinese Longitudinal Healthy Longevity Survey conducted between 2002 and 2018. It included a total of 11,044 Chinese people aged 65 years or older. A group-based trajectory model was used to identify distinctive trajectories of the IADL score, and the Cox proportional hazards model was used to explore the hazard ratio of various trajectories at the onset of MCI. Interaction analysis was used to analyze individual modification between the IADL trajectories and the onset of MCI. Finally, we adopted four types of sensitivity analysis to verify the robustness of the results.

**Results:**

During a median follow-up of 16 years, the incidence of MCI was 6.29 cases per 1,000 person-years (95% confidence interval [CI] 5.92–6.68). Three distinct IADL trajectory groups were identified: a low-risk IADL group (41.4%), an IADL group with increasing risk (28.5%), and a high-risk IADL group (30.4%). Using the Cox proportional hazards model after adjusting for covariates, we found that compared with the low risk IADL group, the hazard ratio of the IADL group with increasing risk was 4.49 (95% CI = 3.82–5.28), whereas that of the high-risk IADL group was 2.52 (95% CI 2.08–3.05). Treating the IADL group with increasing risk as the reference, the hazard ratio for the high-risk IADL group was 0.56 (95% CI 0.48–0.66). Interaction analyses showed that age and residence were significant moderators (*P* for interaction <0.05).

**Conclusion:**

A group-based trajectory model was developed to classify older people into three distinct trajectory groups of the IADL score. The IADL group with increasing risk had a greater risk of MCI than the high-risk IADL group. In the IADL group with increasing risk, city residents of ≥80 years were the most likely to develop MCI.

## 1. Introduction

Mild cognitive impairment (MCI) is an intermediate state between normal aging and dementia, which mostly takes the form of Alzheimer’s disease (1). According to a meta-analysis in China, the prevalence of MCI has reached 12.2% (95% confidence interval [CI] 10.6–14.2) in community residents aged over 55 years (2). To reduce the health burden and enhance the quality of life of older adults, the prevention of dementia is essential (3). Moreover, the onset of MCI is associated with a significant risk of cognitive impairment, which is exhibited by a decline in social activity (4).

Instrumental activities of daily living (IADL) are measured to assess the ability of older adults in independent living, social communication, and completing family tasks (5). If an older adult has lower functioning measured with IADL, it means that their capacity for social activity is seriously hampered (6). Studies on IADL have mainly focused on the prediction and assessment of chronic and critical diseases. The results of a longitudinal cohort study recently indicated that inclusion of IADL impairment in the MCI construct improves the prediction of future dementia (7). Several studies have reported that cognitive impairment and increased age are risk factors for IADL impairment in the social context of China (8–10).

Instrumental activities of daily living impairment and MCI development have been linked through ongoing research. Patients with MCI and dementia have impaired functioning measured with IADL to varying degrees. The functioning in the dementia group was greater impaired than in the MCI group, which is greater than the normal group (*p* < 0.05) (11). With an impairment in cognitive ability, the capacity for complicated social activities shows a dynamic decline, first displayed as a loss in instrumental ability and afterward as an impairment in instrumental activities with lower cognitive requirements (12). This trend has been reported to serve as a dynamic monitoring mechanism for assessing cognitive ability (7), thus providing a new method for predicting the diagnosis of dementia (13). Despite this, some disagreements have arisen in the actual application of the IADL tools, such as in the scoring method (14) and the threshold item division (15). The IADL of older people exhibit complex and varied patterns over time, which add complexity to the study of IADL trajectories.

A group-based trajectory model (GBTM) is an algorithm that can characterize dynamic changes in variables while simultaneously separating a group into multiple trajectory groups and constructing trajectory models within each group (16). In the current study, we used a GBTM to examine and identify relationships and changes within various latent trajectories of IADL.

There is currently a lack of a consistent methodology to accurately identify the risk of MCI. Therefore, we had two main aims in the current study involving longitudinal data from the Chinese Longitudinal Healthy Longevity Survey (CLHLS; 2002–2018): first, to investigate the dynamic IADL trajectories of community-dwelling Chinese elders *via* GBTM, and second, to predict MCI by a Cox proportional hazards model.

## 2. Materials and methods

### 2.1. Study population

#### 2.1.1. Study sample

This research used data from CLHLS. The project was jointly conducted by the Centre for Healthy Aging and Development Studies at Peking University and the Chinese Centre for Disease Control and Prevention. It explored the changes in lifestyle and health status of middle-aged and older people in the changing social environment. The collected information included sociodemographic characteristics, lifestyle, health status, psychological and cognitive status, living environment, and death data.

Eight waves of national surveys have now been conducted by the CLHLS (1998, 2000, 2002, 2005, 2008, 2011, 2014, and 2018). The CLHLS includes 21 provinces, accounting for about 85% of the population of the nation, which makes it the largest longitudinal study of the elderly in developing nations (10). Targeted random sampling is used to select participants in CLHLS. In each province, roughly half of the cities (counties) are chosen to serve as the primary investigative units. To balance the age and sex of older adults, CLHLS uses a multistage stratified random sampling method to follow one nonagenarian, one octogenarian, and three people aged between 65 and 79 years from the same street, village, or town in a primary sampling unit. CLHLS is regarded as a high-quality database because of its robust results of reliability/validity testing, little missing data, and its high response rate (17). Visit the following website to learn details about the CLHLS sampling plan: https://www.icpsr.umich.edu/web/NACDA/studies/36179.

#### 2.1.2. Inclusion and exclusion criteria

For inclusion, participants had to be 65 years or older from the Chinese community, with normal baseline cognitive abilities (based on the clinical diagnosis of dementia) and without dementia or a Mini-Mental State Examination (MMSE) score of ≥24. All participants voluntarily signed the informed consent form in person. Exclusion criteria were a lack of baseline information on living capacity (*n* = 29) and an absence of baseline information on cognitive function measurement (*n* = 2,606).

**Figure 1:**
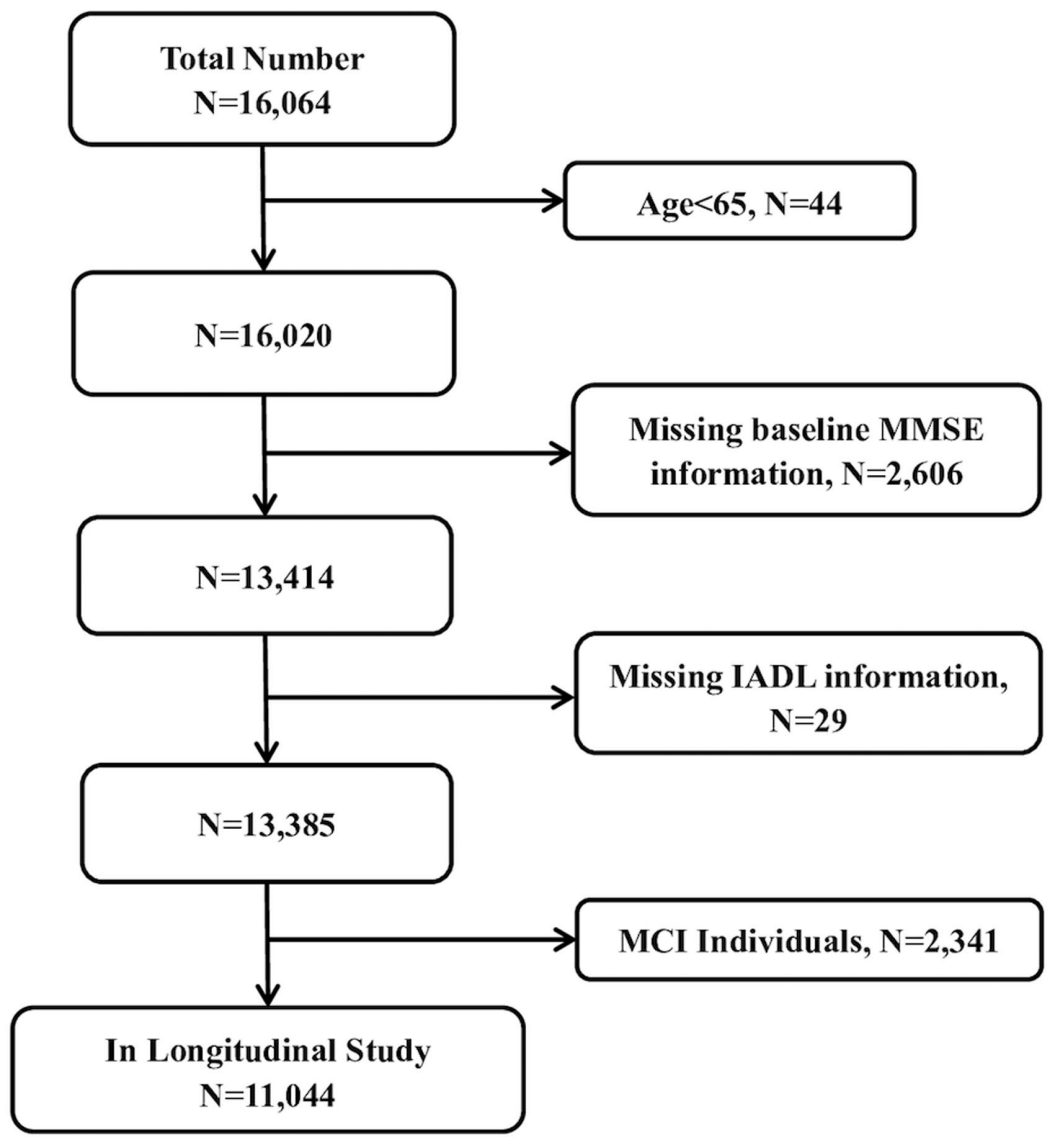
The flow chart of sample selection in the present study. MMSE, Mini Mental State Examination; IADL, instrumental activities of daily living; MCI, mild cognitive impairment.

Investigating the risk prediction of early cognitive impairment based on long-term IADL score changes was necessary for the overall research objective. Consequently, we chose a fixed cohort from 2002, with data available from six waves of investigations. The participants were 11,044 community-dwelling seniors aged 65 years and over (Figure 1).

### 2.2. Measurements

#### 2.2.1. Instrumental activities of daily living

Instrumental activities of daily living was primarily included in this study’s model as a significant independent variable, which measured the health status associated with the social ability of older people in Chinese communities. There are eight sub-items in the IADL, including the following questions: (1) Can you visit your neighbors by yourself? (2) Can you go shopping by yourself? (3) Can you cook a meal by yourself when necessary? (4) Can you wash clothes by yourself when necessary? (5) Can you walk a kilometer at a time by yourself? (6) Can you lift a weight of 5 kg, such as a heavy bag of groceries? (7) Can you continuously squat and stand up three times? (8) Can you take public transportation by yourself? In the CLHLS survey, a score of three negative answers indicates “yes, very limited,” a score of two negative answers denotes “yes, slightly limited,” and one negative answer signifies “not limited.” According to an empirical study, if all the eight indicators of IADL are not limited, it indicates that the elderly individual is fully self-dependent. If one or more items shows that the individual cannot take care of themselves, it suggests that, in respect to IADL, they are disabled (18). The overall score in this study, which ranged from 8 to 24, served as a reference for the participant’s level of IADL disability. The higher the score, the greater the participant’s level of IADL disability (10).

Basic activities of daily living (ADL) reflect the ability of the respondents to live independently. Once ADL are impaired, it means that the patient needs long-term care from nursing staff or family members to ensure their basic living needs. A higher score indicates a greater degree of ADL disability, with an overall score ranging from 6 to 18 points.

#### 2.2.2. Mini-mental state examination

Mini-Mental State Examination has been widely used to assess the cognitive state of older adults. It contains 11 questions related to time and place orientation, reaction, attention, numeracy, memory, and language (19). The participants were required to complete the MMSE questions in person as part of CLHLS to increase the validity of the assessment of cognitive function. A nurse and an investigator assessed the participants’ fundamental cognitive abilities during the evaluation. The question was marked as “unable to answer” if the patient was unable to respond (a score of 0). Higher scores on the MMSE, which range from 0 to 30, indicate better cognitive function (20). As recommended by a geriatric epidemiological survey, MCI was classified by education using the MMSE scale and Petersen criteria (21, 22). For participants who had never received education, an MMSE score of 17 or less was considered as cognitive impairment; for those who had less than 6 years of education, an MMSE score of 20 or less was considered as cognitive impairment; and for those who had more than 6 years of education, an MMSE score of 24 or less was considered as cognitive impairment. For each level of education, scores above the threshold were considered cognitively normal. Beginning in 2002, MMSE was followed up every 2–3 years until MCI occurred or the follow-up period was over.

#### 2.2.3. Covariates

This survey included the collection of sociodemographic and lifestyle factors using a structured questionnaire. The socioeconomic factors included years of schooling (illiterate, primary, and high school), residence (urban and rural), marital status (living without spouse and living with spouse), and income (recoded into tertiles as low, medium, and high). Physical exercise was also divided into three categories, depending on whether or not do it regularly: “never”, “formal”, “present”. Classification variables were also used to describe smoking and drinking status: never, former, and present. Health variables, including physical indicators (age, weight, and chronic disease) and mental indicators (depression), were collected *via* self-reports and objective measurement. The weight of the respondents was measured in kilograms by having them stand on an electronic counting scale after removing their jackets or coats. Chronic diseases, such as hypertension, diabetes, heart disease, pneumonia, and pulmonary tuberculosis, were logged through self-reports. Depression levels were assessed by a series of questions: (1) “Do you always look on the bright side of things?,” (2) “Do you often feel fearful or anxious?,” (3) “Can you make your own decisions concerning your personal affairs?,” (4) “Do you feel the older you get, the more useless you are?,” and (5) “Are you as happy as when you were younger?.” The overall scores ranged from 5 to 25 points, with higher scores indicating lower levels of depression.

### 2.3. Statistical analysis

Stata 16.0 (StataCorp LLC., College Station, TX, United States) was used for descriptive analysis and statistical inference. Continuous variables were described as mean ± standard deviation, whereas categorical variables were expressed as numbers and proportions (%).

We constructed GBTM to identify distinctive IADL trajectory groups and create profiles of the characteristics of these groups. In the analysis, we included all participants who had data on IADL scores collected during six waves from 2002 to 2018. The survey wave was used as a timescale for the trajectories. As a potential class growth model, GBTM was used to analyze longitudinal data and explore heterogeneity. Assuming that there are numerous potential subgroups with various developmental trajectories or patterns in the population, the goal of GBTM is to investigate how many subgroups with various developmental trends are present in the population and to identify the developmental trajectory of each subgroup (16). GBTM predicts the trajectory of each group, the shape of each trajectory, analyzes the individual’s probability of belonging to a group, and places individuals in the group for which they have the highest probability. The first step in GBTM is to determine the number of trajectory groups to include in the model. The fitting effect of the model is reflected in the Bayesian information criterion (BIC) and Akaike information criterion. When the values reach a relative minimum, the best number of trajectory groups is finalized. In addition, the average posterior probability, which reflects the probability of group members belonging to the trajectory, ought to be higher than 0.70 for each group. Moreover, to identify the functional form of the model, each trajectory group is fitted starting from the high-order polynomial to the low-order. If the high-order parameters are not statistically significantly reflected in *p* values or the BIC of the model, the low-order parameters continue to be fitted.

A Cox proportional hazards model was used to investigate the hazard ratios (HRs) of the different trajectories at the onset of MCI, with 95% CIs. Model 1 was adjusted for age and sex; Model 2 was further adjusted for education level, income, marital status, and residence; Model 3 was further adjusted for smoking, alcohol consumption, physical activity, and social activity; and Model 4 was further adjusted for weight, depression, and chronic diseases. Possible modification effects were identified through an interaction effect analysis. The principle of the semiparametric Cox proportional hazards model was to use the product formula to obtain the risk probability related to the baseline risk. The model compensated for the limitations of the univariate Kaplan–Meier survival estimate, which is unable to examine continuous factors (23, 24).

Four distinct sensitivity studies were conducted to confirm the robustness of the results. The first excluded participants who died in the first wave of follow-up. The second excluded participants with baseline chronic diseases. The third involved a multiple interpolation method: a chained equation approach was used to specify the distribution of interpolation variables as the Gaussian normal distribution, while the continuous iterative interpolation method was used to interpolate the missing values. Five sets of data were interpolated, and the regression operation was performed. Finally, the regression coefficients and standard errors of the five sets of regression models were combined (25). In the fourth sensitivity study, a generalized linear mixed model was used. The IADL trajectories were taken as the key independent variable, the MMSE score was taken as the dependent variable, and the individual ID was coded as the second-level variable for model estimation. The hypotheses were tested at a two-sided significance level of *α* = 0.05, and statistical significance was accepted when *p-*values were < 0.05 (two-sided).

## 3. Results

A total of 11,044 respondents (without MCI at baseline) were included in the group-based trajectory analysis (Table 1). During a median follow-up of 16 years, the incidence of MCI was 6.29 (95% CI 5.92–6.68). The average age of the participants was 82.8 ± 11.0 years, and 53.4% (*n* = 5,896) of participants were female. The average weight was 50.4 ± 10.5 kg, 53.0% (*n* = 5,852) of the respondents lived in rural areas, 57.6% (*n* = 6,359) were illiterate, and 37.1% (*n* = 4,096) lived with their spouses. In terms of income, 34.9% (*n* = 3,858) of the respondents had a low income, 33.8% (*n* = 3,727) had a middle income, and 31.3% (*n* = 3,459) had a high income. Additionally, 38.5% (*n* = 4,251) of the participants did physical exercise regularly, but only 14.8% (*n* = 1,640) and 2.4% (*n* = 266) of the participants attended social activities sometimes and often, respectively. In terms of smoking and drinking, 62.8% (*n* = 6,920) had never smoked and 66% (*n* = 7,274) had never consumed alcohol. In terms of mental and physical status, the average depression score was 11.4 ± 3.2, the average IADL score was 11.8 ± 4.9, the average ADL score was 6.4 ± 1.3, and the average MMSE score was 25.6 ± 3.2. Among the 11,044 participants, 34.5% (*n* = 3,809) had one or more chronic diseases.

**Table 1:** The baseline data for participants of CLHLS in 2002.

Variables	Total
	*N* = 11,044
Depression, mean (SD)	11.4 ± 3.2
IADL, mean (SD)	11.8 ± 4.9
ADL, mean (SD)	6.4 ± 1.3
MMSE, mean (SD)	25.6 ± 3.2
Age (years), mean (SD)	82.8 ± 11.0
Weight (kilogram), mean (SD)	50.4 ± 10.5
Sex, *n* (%)
Male	5,148 (46.6%)
Female	5,896 (53.4%)
Years of Schooling, *n* (%)
Illiterate	6,359 (57.6%)
Primary school	3,423 (31.0%)
High school	1,262 (11.4%)
Residence, *n* (%)
Rural	5,852 (53.0%)
City	5,192 (47.0%)
Marital status, *n* (%)
Living without spouse	6,948 (62.9%)
Living with spouse	4,096 (37.1%)
Income, *n* (%)
Low	3,858 (34.9%)
Medium	3,727 (33.8%)
High	3,459 (31.3%)
Smoking, *n* (%)
Never smoking	6,920 (62.8%)
Former smoking	1,807 (16.3%)
Present smoking	2,300 (20.9%)
Drinking, *n* (%)
Never drinking	7,274 (66.0%)
Former drinking	1,287 (11.6%)
Present drinking	2,465 (22.4%)
Social activity, *n* (%)
Never	9,138 (82.8%)
Sometimes	1,640 (14.8%)
Always	266 (2.4%)
Physical activity, *n* (%)
Never	5,825 (52.9%)
Former	952 (8.6%)
Present	4,251 (38.5%)
Chronic disease, *n* (%)
No	7,234 (65.5%)
Yes	3,809 (34.5%)

CLHLS, the Chinese Longitudinal Healthy Longevity Survey; IADL, instrumental activities of daily living; ADL, activities of daily living; MMSE, Mini-Mental State Examination.

**Figure 2:**
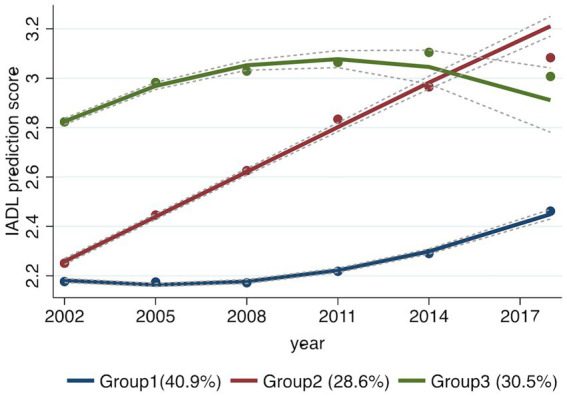
Instrumental activities of daily living (IADl) trajectory groups showing differences across waves. Group 1 named as “low-risk IADL group,” Group 2 named as “IADL group with increasing risk,” and Group3 named as “high-risk IADL group.” IADL, instrumental activities of daily living.

The Akaike information criterion and BIC results showed that the model with three trajectory groups with up to quadratic order terms had the best fit (BIC −7,241.49) and captured the essential features of the data in a more comprehensible and analytically tractable manner (Table 2). Table 3 shows the fitting information of the GBTM, including the testing intercept and linear, quadratic, and cubic specifications for the trajectory shapes. Three distinct trajectories of the community-dwelling Chinese older people were identified (Figure 2). Those in Group 1 (41.1%) who had an IADL score below 2.5 were referred to as the low-risk IADL group. Those in Group 2 (28.5%) who had an IADL score linearly increasing between 2.3 and 3.1 were referred to as the IADL group with increasing risk. Lastly, those in Group 3 (30.4%) who exhibited high levels of IADL between 2.8 and 3.1 during all waves were referred to as the high-risk IADL group.

**Table 2:** Summary information on good-of-fit of IADL trajectory.

Group	AIC	BIC	AvePP		
			Trajectory Group 1	Trajectory Group 2	Trajectory Group 3
1	−11814.78	−11833.05	1		
2	−8630.77	−8660.01	0.918	0.899	
3	−7201.28	−7241.49	0.751	0.801	0.882

AIC, Akaike information criterion; BIC, Bayesian information criterion; AvePP, the average posterior probability.

**Table 3:** Procedure for selecting an IADL trajectory.

Group	Trajectory group	Growth parameter	Est.	*SE*	*T*-value	*p*-value
1	1	Intercept	2.40	0.003	663.47	<0.001
		Linear	0.02	0.003	6.33	<0.001
		Quadratic	−0.003	0.001	−5.21	<0.001
		Cubic	0.0001	0.001	5.49	<0.001
2	1	Intercept	2.19	0.004	586.30	<0.001
		Linear	0.007	0.002	4.49	<0.001
		Quadratic	0.001	0.001	7.61	<0.001
	2	Intercept	2.70	0.006	469.56	<0.001
		Linear	0.05	0.003	18.81	<0.001
		Quadratic	−0.002	0.001	−6.82	<0.001
3	1	Intercept	2.18	0.004	543.47	<0.001
		Linear	−0.01	0.002	−8.34	<0.001
		Quadratic	0.002	0.001	16.93	<0.001
	2	Intercept	2.257	0.006	371.85	<0.001
		Linear	0.06	0.001	59.35	<0.001
	3	Intercept	2.83	0.006	459.36	<0.001
		Linear	0.058	0.004	16.42	<0.001
		Quadratic	−0.003	0.001	−8.01	<0.001

Est., parameter estimate; SE, standard error of parameter estimate.

Table 4 shows the associations between the IADL trajectory groups and the risk of MCI by four Cox proportional hazards models (Group 1 was used as the reference). The HRs were significant in all the models. The HRs for the IADL group with increasing risk and the high-risk IADL group compared with the low-risk IADL group were 4.49 (95% CI 3.82–5.28) and 2.52 (95% CI 2.08–3.05), respectively, as shown in Model 4. Analyzing the sexes separately, the research results of the Cox proportional hazards models were similar to those for the whole group (Supplementary Tables S1, S2). When the IADL group with increasing risk was used as the reference (Supplementary Table S3), the HR for the high-risk IADL group was 0.56 (95% CI 0.48–0.66).

**Table 4:** Cox proportional hazards model for hazard ratio of MCI according to changes in IADL.

Model	IADL Trajectory Group, HR (95%CI)		
	Low-risk IADL group	IADL group with increasing risk	High-risk IADL group
Model 1	1.00	4.44 (3.78–5.20)	2.38 (1.99–2.84)
Model 2	1.00	4.39 (3.74–5.15)	2.41 (2.01–2.89)
Model 3	1.00	4.45 (3.97–5.22)	2.49 (2.07–2.99)
Model 4	1.00	4.49 (3.82–5.28)	2.52 (2.08–3.05)

Model 1 adjusted for age, sex. Model 2 adjusted for variables in Model 1 and education level, income, marital status, and residence. Model 3 adjusted for variables in Model 2 and smoking, alcohol consumption, physical activity, and social activity. Model 4 adjusted for variables in Model 3 and weight, depression, and chronic diseases.

The interaction analysis results showed that age was a significant moderator (*P* for interaction <0.01). Specifically, participants aged 80 years and above in the IADL group with increasing risk (HR 7.49, 95% CI 5.55–10.12, compared with Group 1) were more likely to develop MCI than those younger than 80 years. In addition, when the risk of IADL was increasing, participants living in urban areas had a greater risk of MCI (*P* for interaction <0.05) than rural residents. Treating Group 1 as the reference, in the urban population, the HR of the IADL group with increasing risk was 5.99 (95% CI 4.53–7.92). The onset of MCI was not associated with sex, education level, income, or marital status when controlling for covariates (Table 5).

**Table 5:** Interaction analysis of hazard ratio of IADL trajectory on MCI.

Variables	IADL trajectory group, HR (95%CI)					*P* for interaction
	Low-risk IADL group	IADL group with increasing risk		High-risk IADL group		
**Age (year)**						<0.001
≤80	1.00	3.27	(2.65–4.04)	2.05	(1.44–2.94)	
>80	1.00	7.49	(5.55–10.12)	3.93	(2.91–5.30)	
**Sex**						0.89
Male	1.00	4.61	(3.63–5.85)	2.53	(1.86–3,43)	
Female	1.00	4.39	(3.51–5.49)	2.49	(1.94–3.20)	
**Years of schooling**						0.18
Illiterate	1.00	3.90	(3.14–4.84)	2.42	(1.90–3.07)	
Primary school	1.00	5.18	(3.84–6.98)	2.69	(1.85–3.92)	
High school	1.00	5.82	(3.80–8.91)	2.35	(1.26–4.38)	
**Residence**						0.02
Rural	1.00	3.81	(3.12–4.66)	2.36	(1.87–2.99)	
City	1.00	5.99	(4.53–7.92)	2.85	(2.06–3.94)	
**Income**						
Low	1.00	4.03	(3.16–5.13)	2.50	(1.88–3.33)	0.40
Medium	1.00	4.29	(3.22–5.71)	2.17	(1.55–3.04)	
High	1.00	5.85	(4.15–8.25)	3.22	(2.17–4.80)	
**Marital status**						0.27
Never married	1.00	4.99	(4.01–6.21)	2.79	(2.20–3.53)	
Married	1.00	3.78	(2.94–4.86)	2.21	(1.54–3.16)	

HR (95%CI) adjusted for age, sex, years of schooling, income, marital status, smoking, alcohol consumption, physical activity, social activity, weight, depression, and chronic diseases (remove grouping variables). HR, hazards ratio.

Four sensitivity analysis protocols were also conducted (Table 6). With the multiple interpolation method, the HR in the IADL group with increasing risk was 4.46-fold higher than that in the low-risk IADL group (HR 4.46, 95% CI 3.79–5.24). The HR of MCI in the high-risk IADL group was 2.48-fold higher than that in the low-risk IADL group (HR 2.48, 95% CI 2.05–3.00). After participants who passed away during the first wave of follow-up were excluded, the adjusted HR was 3.13 (95% CI 2.65–3.70) for the IADL group with increasing risk and 3.15 (95% CI 2.60–3.82) for the high-risk IADL group. After eliminating participants with chronic disease at baseline, the adjusted HRs in the IADL group with increasing risk and the high-risk IADL group were 4.55 (95% CI 3.75–5.23) and 2.59 (95% CI 2.05–3.27), respectively, compared with the low-risk IADL group. The average MMSE score for the IADL group with increasing risk was 1.33-fold lower than that of the low-risk IADL group (95% CI −1.47, −1.19), and for the high-risk IADL group it was 2.29-fold lower (95% CI −2.45, −2.13) than that of the low-risk IADL group.

**Table 6:** Sensitivity analysis of hazard risks of IADL trajectory on MCI.

IADL trajectory groups	Model 1, HR (95% CI)^a^	Model 2, HR (95% CI)^b^	Model 3, HR (95% CI)^c^	Model 4, *β* (95% CI)^d^
Low-risk IADL group	1	1	1	1
IADL group with increasing risk	4.46 (3.79,5.24)	3.13 (2.65,3.70)	4.55 (3.75,5.23)	−1.33 (−1.47, −1.19)
High-risk IADL group	2.48 (2.05,3.00)	3.15 (2.60,3.82)	2.59 (2.05,3.27)	−2.29 (−0.2.45, −2.13)

^a^Multivariate Cox regression results after multiple interpolation; ^b^Multivariate Cox regression results after excluding patients who died in the first circle; ^c^Cox regression results excluding patients with chronic diseases at baseline; ^d^Adopt the generalized mixed-effect model. Covariates such as age, sex, education level, income, marital status, smoking, alcohol consumption, physical activity, social activity, weight, depression, and chronic diseases were adjusted for all sensitivity analyses. HR, hazard ratio.

## 4. Discussion

To the best of our knowledge, this was the first study to divide IADL into three trajectory groups in an older Chinese community-dwelling population to investigate the relationship between IADL trajectories and the onset of MCI. The IADL group with increasing risk and the high-risk IADL group both had greater risks of developing MCI than the low-risk IADL group. The IADL group with increasing risk had the highest risk of developing MCI. In adults over 80 years of age living in cities, the risk of MCI rose with increasing IADL impairment, according to the interaction analysis.

In the CLHLS, the prevalence of MCI was 17.5% in the Chinese community aged 65 years and over at baseline. According to previous surveys in China, the prevalence of MCI (using the Petersen criteria) ranged from 11.33 to 20.80% among individuals of 65 years of age and older (26–28). Although the survey results show heterogeneity as a result of various research designs, social background differences, and sampling errors, a Chinese meta-analysis reported the combined prevalence of MCI in adults over 55 years at 12.2% (2). The findings reveal a latent MCI trend in Chinese communities. Therefore, effective assessment tools and MCI prevention strategies are necessary.

Group-based trajectory model was used to classify IADL into three distinctive trajectory groups in this longitudinal study: the low-risk IADL group, the IADL group with increasing risk, and the high-risk IADL group. Most previous studies on the evolution of older people’s daily living skills have focused on IADL. A cohort study conducted in Chinese community-living older people found that IADL trajectories either showed a sharp decline from a high starting point or a rapid decline from a low starting point (29). We included groups with increasing risk (linear change) and static high-risk or low-risk (constant level) related to IADL during follow-up. Among the three trajectory groups, the HRs for MCI were highest for the IADL group with increasing risk, intermediate for the high-risk IADL group, and lowest for the low-risk IADL group. Similar conclusions were made in a UK health and retirement study, which was based on a latent growth trajectory model. It reported that in middle-aged people (50–64 years), worse ADL and IADL outcomes were closely associated with cognitive impairment (non-dementia) and predicted dementia in later life (30).

What are the underlying mechanisms that explain why the IADL group with increasing risk had the highest MCI risk among the three trajectory groups? The disability of the individuals in the high-risk IADL group cannot be ignored, and thus corresponding nursing and medical measures are taken promptly. By contrast, the performance of the individuals in the IADL group with increasing risk might be hidden by functional compensation, making disability harder to recognize. The “disability paradox” claims that senior citizens with self-reported severe disability still report high quality of life even though the disability is linked to higher healthcare costs, premature death, and impaired quality of life (31–33). Therefore, the “paradox” is influenced by the social context and external environment of the individual (34).

Dynamic switching between different disability states can occur in older people. For example, a multimodal model of disability transition among Chinese older people was developed to analyze the transition rate of four modes: no disability, mild disability, severe disability, and death. According to this study, aging significantly reduced the rate of change from a disabled to a non-disabled status (35). From the perspective of social stratification, rural areas had a higher rate of mild disability rehabilitation than urban areas (35). The transition to severe disability was more common than improvement among individuals older than 85 years of age (36).

We found that in the IADL group with increasing risk, individuals over 80 years of age and those living in the city had a higher risk of developing MCI than those under 80 and those living in rural areas (*p* < 0.05). In China’s rural areas, the standard of medical and health care is lower, and high-quality medical care facilities are more sparsely concentrated (37). Senior residents in rural areas also tend to have less medical knowledge, which could contribute to a shorter life span than their urban counterparts (38). In the absence of health education and exercise facilities, rural residents have a low self-reported rate of regular physical exercise (39). However, the rural residents in our study had a significantly lower risk of MCI than urban residents in the IADL group with increasing risk, indicating that the environment had an impact on the disability of these community elders. An underlying mechanism related to “survival choice” needs to be taken into consideration. Owing to the poorer access to medical services in rural areas, older people who are frail in these areas may die prematurely, whereas the older people who survive may have some stronger characteristics (such as genes and behaviors) against disability. This process leads to regional inequality in MCI related to IADL disability (40).

Increasing age was identified as an essential factor regulating IADL from an individual perspective, consistent with the findings of previous studies. Elderly people have weaker immune systems, are less physically active, and have less capacity for recovery compared with younger older people, which reduces their chances of recovering from injuries or illnesses (41). Moreover, a “male–female health-survival paradox” has been reported in which male people typically have fewer disabilities than female people but have shorter lives (42). In the sensitivity analyses, to limit the effect of the choice paradox, we excluded participants with chronic diseases at baseline or those who had died by the first follow-up to confirm that the results were still robust. This supported our findings that IADL impairment increased the risk of MCI and that this risk was higher for the IADL group with increasing risk than for the high-risk IADL group.

Our study had several strengths. First, we used GBTM to classify older individuals into three distinct trajectory groups of the IADL score to examine the risk of MCI in these different groups. To our knowledge, this is the first study to document the connection between the trajectory of IADL and MCI risk. We assessed the effect of the IADL trajectory on MCI using the Cox proportional hazards model. The long-term follow-up from 2002 to 2008 and the sizable sample size offered adequate statistical power. Furthermore, four types of sensitivity analyses were used to confirm that the IADL trajectory estimations were reliable as indicators of MCI risk.

There were some limitations to our study. First, as the participants were older individuals, there were deaths during the follow-up period, resulting in loss of some sample information to estimate the model. Most variables in the present study were obtained by self-reported questionnaires, especially in terms of information on chronic diseases. More physical examinations and laboratory objective indicators should be considered in future to reduce the Hawthorne effect. In addition, five depression-related questions were self-compiled by CLHLS investigators to assess the depressive status of the respondents before 2018; thus, a complete depression scale was lacking. Finally, the MMSE scale was used to measure MCI. Although this method has been verified in population studies, it is not a method used in clinical diagnoses. Some objective means, such as molecular targets and iconography methods, may be more helpful in clarifying the diagnosis of MCI.

## 5. Conclusion

A GBTM was developed to classify community-dwelling Chinese seniors into three distinct trajectory groups of the IADL score. The participants’ age and place of residence had various effects on how IADL impairment affected MCI incidence. Individuals of ≥80 years of age living in urban rather than rural locations in the IADL group with increasing risk were the most likely to develop MCI. Our study provides evidence for monitoring IADL change in older adults. In terms of MCI management, the findings underline the need for basic medical and health services for older people living in cities.

## Data availability statement

The original contributions presented in the study are included in the article/Supplementary material, further inquiries can be directed to the corresponding authors.

## Ethics statement

All the procedures used to support this work were carried out in agreement with the necessary standards and laws. The datasets used to support this work are available to the public through the CLHLS project. It received approval from Peking University’s research ethics committees (IRB00001052–13074). All the participants signed a written informed consent.

## Author contributions

JY, YCZ, LW, and XL designed the study. SS, HY, LY, YOZ, YX, and JS collected and systematized the data. YCZ analyzed the data. JY drafted the manuscript. LW and XL polished the manuscript. All authors contributed to the article and have approved the submitted version.

## Funding

The present study was supported by the National Natural Science Foundation of China (Grant No. 72174033) and the Science and Technology Research Project of Chongqing Education Commission (Grant No. KJQN202100432).

## Conflict of interest

The authors declare that the research was conducted in the absence of any commercial or financial relationships that could be construed as a potential conflict of interest.

## Publisher’s note

All claims expressed in this article are solely those of the authors and do not necessarily represent those of their affiliated organizations, or those of the publisher, the editors and the reviewers. Any product that may be evaluated in this article, or claim that may be made by its manufacturer, is not guaranteed or endorsed by the publisher.
