# Supplementary material for: Instrumental activities of daily living trajectories and risk of mild cognitive impairment among Chinese older adults: results of the Chinese longitudinal healthy longevity survey, 2002–2018
Source: Front Public Health. 2023 May 3;11:1165753. doi: 10.3389/fpubh.2023.1165753 (PMC10189058; doi:10.3389/fpubh.2023.1165753)
Supplement: Supplementary file 1 [file Table_1.DOCX]

**Supplementary Table S1.** Cox Proportional Hazards model for hazard ratio of MCI according to changes in IADL for Male

| Model | IADL Trajectory Group, HR (95%CI) | | |
| --- | --- | --- | --- |
|  | Low-risk IADL group | IADL group with  increasing risk | High-risk IADL group |
| Model 1 | 1.00 | 4.49 (3.55 - 5.66) | 2.18 (1.64 - 2.90) |
| Model 2 | 1.00 | 4.43 (3.51 - 5.60) | 2.33 (1.75 - 3.11) |
| Model 3 | 1.00 | 4.53 (3.58 - 5.73) | 2.42 (1.80 - 3.26) |
| Model 4 | 1.00 | 4.61 (3.63 - 5.85) | 2.53 (1.86 - 3.43) |
| **Notes:** Model 1 adjusted for age. Model 2 adjusted for variable in Model 1 and education level, income, marital status, and residence. Model 3 adjusted for variables in Model 2 and Smoking, alcohol consumption, physical activity, and social activity. Model 4 adjusted for variables in Model 3 and Weight, depression, and chronic diseases. | | | |

**Supplementary Table S2.** Cox Proportional Hazards model for hazard ratio of MCI according to changes in IADL for Female

| Model | IADL Trajectory Group, HR (95%CI) | | |
| --- | --- | --- | --- |
|  | Low-risk IADL group | IADL group with  increasing risk | High-risk IADL group |
| Model 1 | 1.00 | 4.44 (3.57 - 5.52) | 2.48 (1.96 - 3.14) |
| Model 2 | 1.00 | 4.36 (3.50 - 5.42) | 2.44 (1.92 - 3.10) |
| Model 3 | 1.00 | 4.41 (3.54 - 5.49) | 2.51 (1.97 - 3.20) |
| Model 4 | 1.00 | 4.39 (3.51 - 5.49) | 2.49 (1.94 - 3.20) |
| **Notes:** Model 1 adjusted for age. Model 2 adjusted for variable in Model 1 and education level, income, marital status, and residence. Model 3 adjusted for variables in Model 2 and Smoking, alcohol consumption, physical activity, and social activity. Model 4 adjusted for variables in Model 3 and Weight, depression, and chronic diseases. | | | |

**Supplementary Table S3.** Cox Proportional Hazards model for hazard ratio of MCI according to changes in IADL (Treating Group 2 as the Reference)

| Model | IADL Trajectory Group, HR (95%CI) | | |
| --- | --- | --- | --- |
|  | Low-risk IADL group | IADL group with  increasing risk | High-risk IADL group |
| Model 1 | 0.23 (0.19 - 0.26) | 1.00 | 0.54 (0.46 - 0.62) |
| Model 2 | 0.23 (0.19 - 0.27) | 1.00 | 0.55 (0.47 - 0.64) |
| Model 3 | 0.22 (0.19 - 0.26) | 1.00 | 0.56 (0.48 - 0.65) |
| Model 4 | 0.22 (0.19 - 0.26) | 1.00 | 0.56 (0.48 - 0.66) |
| **Notes:** Model 1 adjusted for age, sex. Model 2 adjusted for variables in Model 1 and education level, income, marital status, and residence. Model 3 adjusted for variables in Model 2 and Smoking, alcohol consumption, physical activity, and social activity. Model 4 adjusted for variables in Model 3 and Weight, depression, and chronic diseases. | | | |
